# Negative regulation of TGFβ-induced apoptosis by *RAC1B* enhances intestinal tumourigenesis

**DOI:** 10.1038/s41419-021-04177-7

**Published:** 2021-09-25

**Authors:** Victoria Gudiño, Patrizia Cammareri, Caroline V. Billard, Kevin B. Myant

**Affiliations:** 1 Cancer Research UK Edinburgh Centre, MRC Institute of Genetics & Molecular Medicine, The University of Edinburgh, Western General Hospital, Crewe Road South, Edinburgh, EH4 2XU UK; 2 Present Address: Inflammatory Bowel Disease Unit, Department of Gastroenterology, Institut d’Investigacions Biomèdiques August Pi i Sunyer (IDIBAPS) - CIBEREHD, Barcelona, Spain

**Keywords:** Cancer genetics, Apoptosis

## Abstract

*RAC1B* is a tumour-related alternative splice isoform of the small GTPase *RAC1*, found overexpressed in a large number of tumour types. Building evidence suggests it promotes tumour progression but compelling in vivo evidence, demonstrating a role in driving tumour invasion, is currently lacking. In the present study, we have overexpressed *RAC1B* in a colorectal cancer mouse model with potential invasive properties. Interestingly, *RAC1B* overexpression did not trigger tumour invasion, rather it led to an acceleration of tumour initiation and reduced mouse survival. By modelling early stages of adenoma initiation we observed a reduced apoptotic rate in *RAC1B* overexpressing tumours, suggesting protection from apoptosis as a mediator of this phenotype. *RAC1B* overexpressing tumours displayed attenuated TGFβ signalling and functional analysis in ex vivo organoid cultures demonstrated that *RAC1B* negatively modulates TGFβ signalling and confers resistance to TGFβ-driven cell death. This work defines a novel mechanism by which early adenoma cells can overcome the cytostatic and cytotoxic effects of TGFβ signalling and characterises a new oncogenic function of RAC1B in vivo.

## Introduction

Colorectal cancer (CRC) is the second leading cause for cancer-related deaths worldwide [1]. The genetic alterations and signalling pathways involved during the formation and progression of CRC include the Wnt signalling pathway, RAS/mitogen-activated protein kinase (MAPK), transforming growth factor beta (TGFβ) and *TP53* signalling [2]. *TP53* is mutated in about 50% of all CRC tumours [3] and the Vogelstein model classifies its loss of function as a late event during the adenoma-carcinoma sequence [4]. In genetically modified mice, deletion of *TP53* is insufficient to initiate intestinal tumourigenesis. However, loss of *TP53* in cooperation with other cancer driver events such as *Apc* loss, or treatment with Azoxymethane (AOM), a chemical inducer of *Ctnnb1* mutations, enhances tumorigenesis and invasion [5, 6]. This suggests that concomitant *TP53* deletion and activation of the Wnt signalling pathway might be a suitable model for the study of invasive intestinal tumours.

Rac1 is a small Rho GTPase that acts as a molecular switch for multiple signalling cascades [7]. Whilst its expression is ubiquitous across all tissues, its activation is context and time-specific controlled by guanine nucleotide exchange factors (GEFs), GTP-activating proteins (GAPs) and guanine nucleotide dissociation inhibitors (GDIs) [8]. Rac1 activity is also regulated through its alternative spliced isoform *RAC1B*. *RAC1B* results from the in-frame insertion of exon 3b, which is usually skipped during splicing [9]. This insertion prevents GTP hydrolysis and binding to GDIs resulting in a constitutively GTP-bound, active state [10].

High Rac1 activity is associated with tumourigenic processes, such as cell adhesion, invasion promotion and stem cell expansion [11–13]. Hence, constitutively active RAC1B can activate the pathway and drive cellular transformation. The first indication for its tumour-associated role lies with its preferential expression in tumours compared to healthy tissue [14–16]. This is particularly apparent in CRC and we have recently shown using the The Cancer Genome Atlas (TCGA) database that high *RAC1B* expression associates with late stage, metastatic disease and poor patient prognosis [17]. This is in line with previous investigations that have demonstrated an invasive function for RAC1B. For instance, it was shown that MMP3 promotes epithelial-to-mesenchymal transition (EMT) through hnRNP A1-dependent *RAC1B* production [18, 19]. Likewise, RAC1B is necessary for EMT-bypass of senescence in lung adenocarcinoma [20]. Other suggested mechanisms driven by RAC1B to promote invasion and EMT include downregulation of E-cadherin and/or interaction with the GAP protein ArhGAP11A [21, 22]. Emerging in vivo evidence indicates an oncogenic role of RAC1B. Zhou et al. showed that overexpression of *RAC1B* in a K-ras-driven lung adenocarcinoma model enhanced tumorigenesis [23] and similarly, Kotelevets et al. recently demonstrated cooperation between RAC1B and the Wnt signalling pathway to promote intestinal tumour formation [24]. We have recently demonstrated a role for RAC1B in modulating oncogenic EGFR and WNT signalling in intestinal tumourigenesis [17]. However, except for Stallings-Mann and colleagues work using a lung adenocarcinoma mouse model [20], in vivo validation of a role for RAC1B in promoting tumour invasion is currently lacking.

To determine whether *RAC1B* overexpression promotes tumour invasion in vivo, we have developed an intestinal-specific mouse model whereby *RAC1B* is overexpressed, along with *Apc* and *TP53* deletion. Surprisingly, these mice presented a tumour initiation rather than a tumour invasion phenotype, as overexpression of *RAC1B* led to a significantly reduced survival due to increased number of intestinal tumours. Mechanistically, RAC1B conferred resistance to TGFβ-induced cell death through downregulation of core TGFβ pathway genes and the pro-apoptotic target gene *Bim*. Moreover, we observed an association between mutations in the TGFβ signalling pathway and *RAC1B* expression in human tumours samples from the TCGA database, suggesting that RAC1B might act as a non-mutational mechanism for attenuating the anti-tumorigenic functions of the TGFβ pathway in tumour initiating cells.

## Results

### *RAC1B* overexpression increases intestinal tumour initiation

**Fig. 1:**
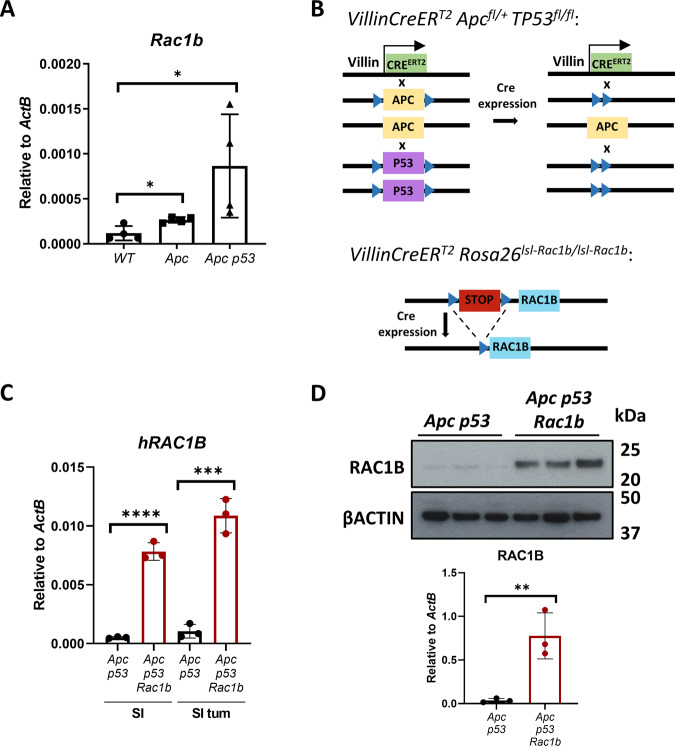
Generating a mouse tumour model with *RAC1B* overexpression. **A** qRTPCR analysis of mouse *Rac1b* in the small intestine of wild-type (*WT*) mice, and in tumours from aged *Apc*^*fl/+*^ (*Apc*) and *Apc*^*fl/+*^ *p53*^*fl/fl*^ (*Apc p53*) mice (error bars represent SD; *P < 0.05; two tailed *t* test, *n* = 4vs4vs4). **B** Schematic outlining the genetic strategy to delete *APC* and *TP53* and to overexpress *RAC1B*. **C** qRT-PCR analysis of *hRAC1B* in small intestine (SI) tumours and matched normal tissue of *Apc p53* and *Apc p53 Rac1b* mice (errors bars represent SD; ***P < 0.001, ****P < 0.0001; two tailed *t* test, *n* = 3vs3). **D** Western blot analysis of intestinal epithelial extractions of *Apc p53* and *Apc p53 Rac1b* mice for RAC1B expression. Graph showing RAC1B band densitometry quantification relative to βACTIN is presented below (error bars represent SD; **P < 0.01; two tailed *t* test, *n* = 3vs3).

**Fig. 2:**
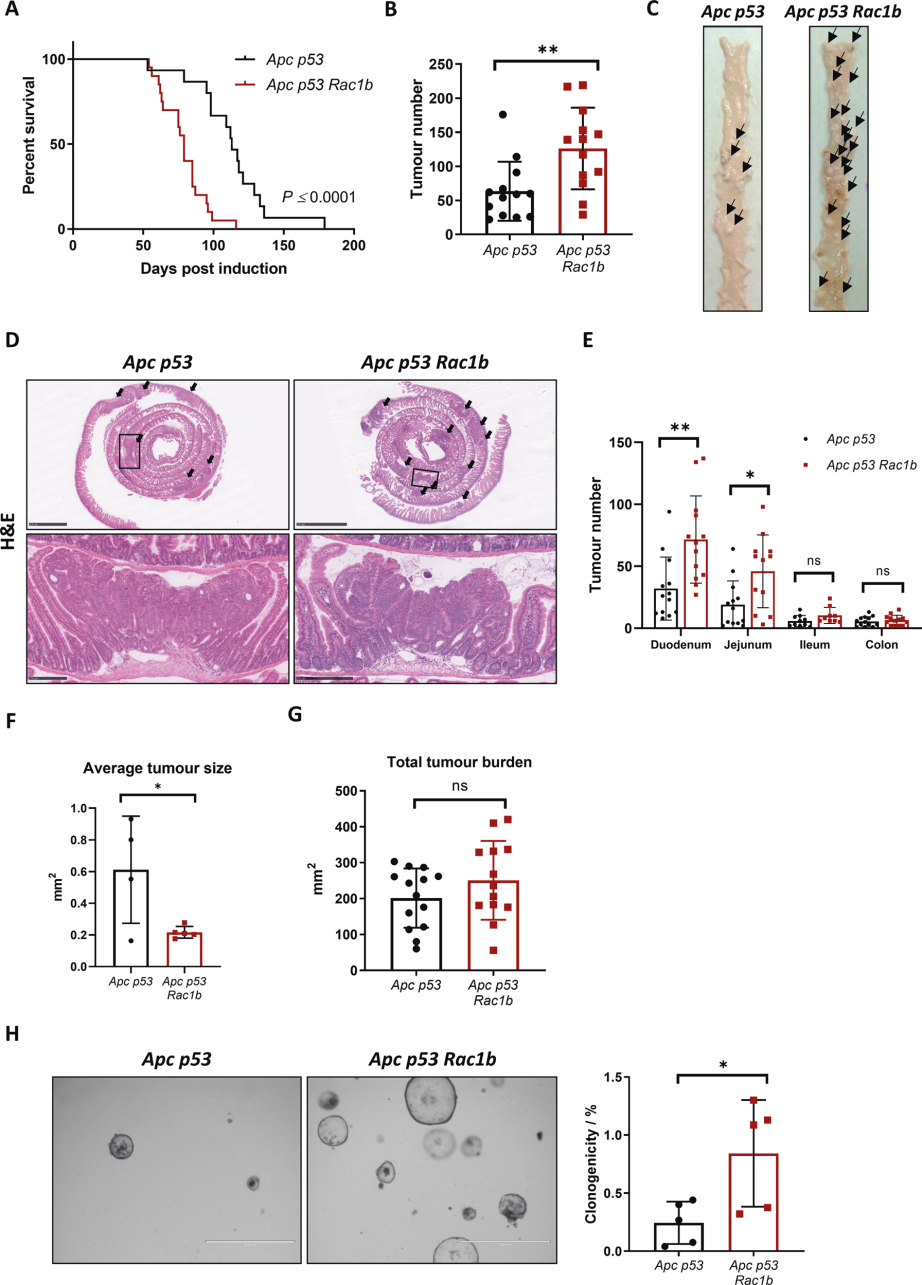
Overexpression of *RAC1B* promotes tumourigenesis and decreases mouse survival. **A** Survival plot of *Apc p53* vs *Apc p53 Rac1b* mice. Log-Rank ****P = 0.000019, *n* = 15vs20). **B** Quantification of total tumour number in the intestine of *Apc p53* and *Apc p53 Rac1b* mice (errors bars represent SD; **P = 0.0054; two tailed *t* test, *n* = 13vs13). **C** Representative images of intestines from *Apc p53* and *Apc p53 Rac1b* mice. Black arrows indicate the presence of intestinal tumours. **D** H&E of representative whole small intestinal rolls from *Apc p53* and *Apc p53 Rac1b* mice. Black arrows indicate the presence of tumours and boxes indicate area of magnified image below. Scale bars are 2.5 mm (top) and 250 µm (bottom). **E** Quantification of total number of tumours grouped based on the intestinal region (duodenum, jejunum, ileum and colon) of *Apc p53* and *Apc p53 Rac1b* mice (error bars represent SD; **P < 0.01, *P < 0.05, #*P* < 0.1, *P* ≥ 0.05; two tailed *t* test, *n* = 13vs13). **F** Quantification of the average size of tumours from histological sections from *Apc p53* and *Apc p53 Rac1b* mice (errors bars represent SD; *P < 0.05; two tailed *t* test; *n* = 4vs5). **G** Quantification of the total area of tumours (tumour burden) in the intestines of *Apc p53* and *Apc p53 Rac1b* mice (error bars represent SD; *P* ≥ 0.05; two tailed *t* test, *n* = 13vs13). **H** Clonogenicity assay of *Apc p53* and *Apc p53 Rac1b* mousederived organoids. Images are representative pictures and graph represents the percentage of clonogenicity per each cell line. Scale bars are 1000 µm (error bars represent SD; *P = 0.027; two tailed *t* test, *n* = 5vs5).

We previously found increased expression of *RAC1B* in CRC correlates with advanced tumour stage [17]. Likewise, analysis of tumours derived from various CRC mouse models showed that *Rac1b* expression increases as mice accumulate additional mutations, emulating more aggressive human tumours (Fig. 1A). To investigate the functional implication of *RAC1B* overexpression in CRC, we used a mouse model with intestine-specific deletion of *Apc* and *TP53* (*VillinCreER*^*T2*^ *Apc*^*fl/+*^ *TP53*^*fl/fl*^, referred from now on as *Apc p53*). A recent study has demonstrated that mice carrying these mutations present with occasional invasive tumours suggesting this is a suitable model for determining the impact of additional alterations on promoting tumour invasion (Fig. 1B) [25]. To test whether overexpression of *RAC1B* promotes tumour invasion, we crossed *Apc p53* mice with mice carrying an inducible overexpression allele of human *RAC1B* in the *Rosa26* locus (*VillinCreER*^*T2*^ *Apc*^*fl/+*^ *TP53*^*fl/fl*^ *Rosa26*^*lsl-hRAC1B/lsl-hRAC1B*^, referred to from now on as *Apc p53 Rac1b*) (Fig. 1B). Cohorts of *Apc p53* and *Apc p53 Rac1b* mice were induced with tamoxifen and aged until they became symptomatic of disease (pale feet, weight loss and hunching). Expression of *hRAC1B* in *Apc p53 Rac1b* mice was confirmed by qRT-PCR and Western blot (Fig. 1C, D). Interestingly, *Apc p53 Rac1b* mice started to manifest endpoint clinical signs ~60 days post induction, whereas control mice were not disease symptomatic until ~90 days post induction. Consequently, *RAC1B* overexpressing mice survived to a median of 79 days, while control mice survived to a median of 113 days (Fig. 2A). Macroscopic scoring of the intestinal tumours revealed that *Apc p53 Rac1b* mice developed significantly more tumours than *Apc p53* controls (Fig. 2B, C), which were mainly localised in the small intestine (Fig. 2D, E). However, none of these tumours showed signs of invasion, suggesting that increased tumour number rather than a more aggressive tumour stage was the cause for their early endpoint. Moreover, the rapid tumour formation observed in the *Apc p53 Rac1b* cohort led to the development of significantly smaller tumours compared to controls (Fig. 2F). Of note, tumour burden, which measures the total area of tumours per mouse, was similar in both genotypes, indicating both cohorts were terminated at the same clinical endpoints (Fig. 2G). Histopathological analysis of *Apc p53* and *Apc p53 Rac1b* tumours did not reveal significant differences between the two groups. Tumours showed similar levels of differentiated and proliferative cells as detected by Periodic Acid-Shiff (PAS) and BrdU immunohistochemistry (IHC), respectively (Fig. S1A, B). In terms of cell death, there were no significant differences in the number of cleaved caspase-3 (CC3) positive cells between genotypes, although there seemed to be a trend towards a reduced percentage of apoptosis in the *RAC1B* overexpressing mice (*p* = 0.071, Fig. S1C). To better measure the intrinsic ability of epithelial cells to promote intestinal tumorigenesis, we digested *Apc p53* and *Apc p53 Rac1b* tumours into single cells and grew them as 3D organoids to evaluate their clonogenic potential in vitro. Tumours bearing *RAC1B* overexpression presented a significantly enhanced clonal capacity compared to the *Apc p53* control (Fig. 2H). This demonstrates that overexpression of *RAC1B* confers a higher colony forming capacity on tumour cells, which might facilitate their tumourigenesis in vivo. Overall, these data suggest that overexpression of *RAC1B* in cooperation with *Apc* and *TP53* deletion promotes intestinal tumour initiation.

### Early adenomas with RAC1B overexpression are less apoptotic

To investigate early cell transformation changes driven by RAC1B that might lead to the observed enhanced tumorigenesis, we designed a pre-tumorigenic mouse model strategy. *Apc p53* and *Apc p53 Rac1b* mice were induced and sampled 31 days post induction, which is approximately half the average survival for the *Apc p53 Rac1b* group.

**Fig. 3:**
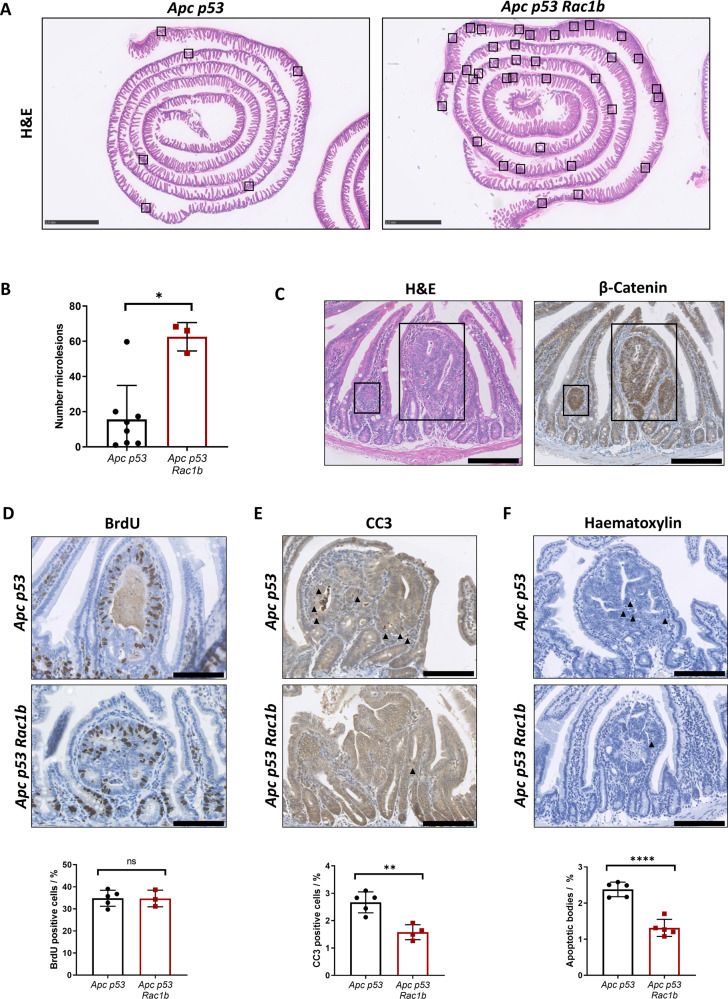
Early adenoma lesions with *RAC1B* overexpression contain less apoptotic cells. **A** Representative H&E of part of the small intestine from *Apc p53* and *Apc p53 Rac1b* mice 31 days post tamoxifen induction. Black boxes highlight micro lesions location. Scale bars are 2.5 mm. **B** Quantification of microlesions from the small intestines of *Apc p53* vs *Apc p53 Rac1b* mice (error bars represent SD; *P = 0.024; two tailed Mann–Whitney test, *n* = 8vs3). **C** Serial sections of a representative microlesion stained with H&E and βCatenin IHC. Black boxes delimit microlesions. Scale bars are 250 µm. **D** BrdU IHC of microlesions from *Apc p53* and *Apc p53 Rac1b* mice. Percentage of proliferation is shown based on the number of BrdU positive cells relative to the total number of cells within the microlesion. Scale bars are 100 µm (error bars represent SD; *P* ≥ 0.05; two tailed *t* test, *n* = 5vs3). **E** CC3 IHC of microlesions from *Apc p53* and *Apc p53 Rac1b* mice. Percentage of apoptosis is shown based on the number of CC3 positive cells relative to the total number of cells within the microlesion. Scale bars are 100 µm (error bars represent SD; **P = 0.002; two tailed *t* test, *n* = 5vs4). **F** Haematoxylin staining of micro lesions from *Apc p53* and *Apc p53 Rac1b* mice. Percentage of apoptosis is shown based on the number of apoptotic bodies (AB) relative to the total number of cells within the microlesion. Scale bars are 100 µm (error bars represent SD; ****P < 0.0001; two tailed *t* test, *n* = 5vs5).

Interestingly, we noticed a number of microadenoma lesions in the intestinal sections from these mice (Fig. 3A). Quantification analysis from Swiss roll sections demonstrated that *Apc p53 Rac1b* mice developed significantly more lesions than *Apc p53* controls, indicating that the enhanced tumour initiation driven by *RAC1B* overexpression is already detectable at early time points (Fig. 3B). To confirm that these lesions were early adenomas which had lost *Apc* heterozygosity, sections were stained for β-catenin. All lesions presented nuclear β-catenin localisation, characterising them as microlesions or early adenomas (Fig. 3C).

There are a number of mechanistic reasons that could explain the increased tumourigenesis seen in both models. For example, RAC1B might enhance epithelial proliferation through activation of the Wnt signalling pathway [17, 21]. However, overexpression of *RAC1B* did not increase the number of proliferative cells in the normal small intestine nor in the early adenomas (Figs. 3D and S2A) and did not increase the expression of canonical Wnt target genes such as *Axin2, Tcf7, Lef1 or Ctnnb1* (Fig. S2B). Alternatively, RAC1B could promote the loss of *Apc*, but β-catenin staining of the normal epithelium showed the same nuclear localisation pattern in both genotypes (Fig. S2C). We also tested whether RAC1B might have expanded the stem cell population or altered cellular differentiation, which could suggest tumourigenesis with villus origin. However, both stem cell (*Lgr5, Olfm4 and Slc14a1*) and differentiated cell markers (*Lyz1, Defa20 and Muc2*) presented a similar expression between genotypes (Fig. S2B, C). Finally, RAC1B did not lead to enhanced DNA damage as both groups show a similar pattern of pH2AX staining (Fig. S2C). We then investigated whether changes in cell death might explain RAC1B-driven tumourigenesis. Staining of cleaved-caspase3 (CC3) in these lesions showed that mice with *RAC1B* overexpression presented a significantly reduced percentage of CC3 positive cells compared to controls (Fig. 3E). Likewise, quantification of apoptotic bodies in haematoxylin-stained sections, further validated this result (Fig. 3F).

Overall, this suggests that enhanced tumour initiation observed in both pretumorigenic and ageing tumour models driven by *RAC1B* overexpression may be a result of a reduced apoptotic rate, which in turn allows the survival of transformed cells and subsequent tumour formation.

### RAC1B protects from cell death via downregulation of the TGFβ pathway

**Fig. 4:**
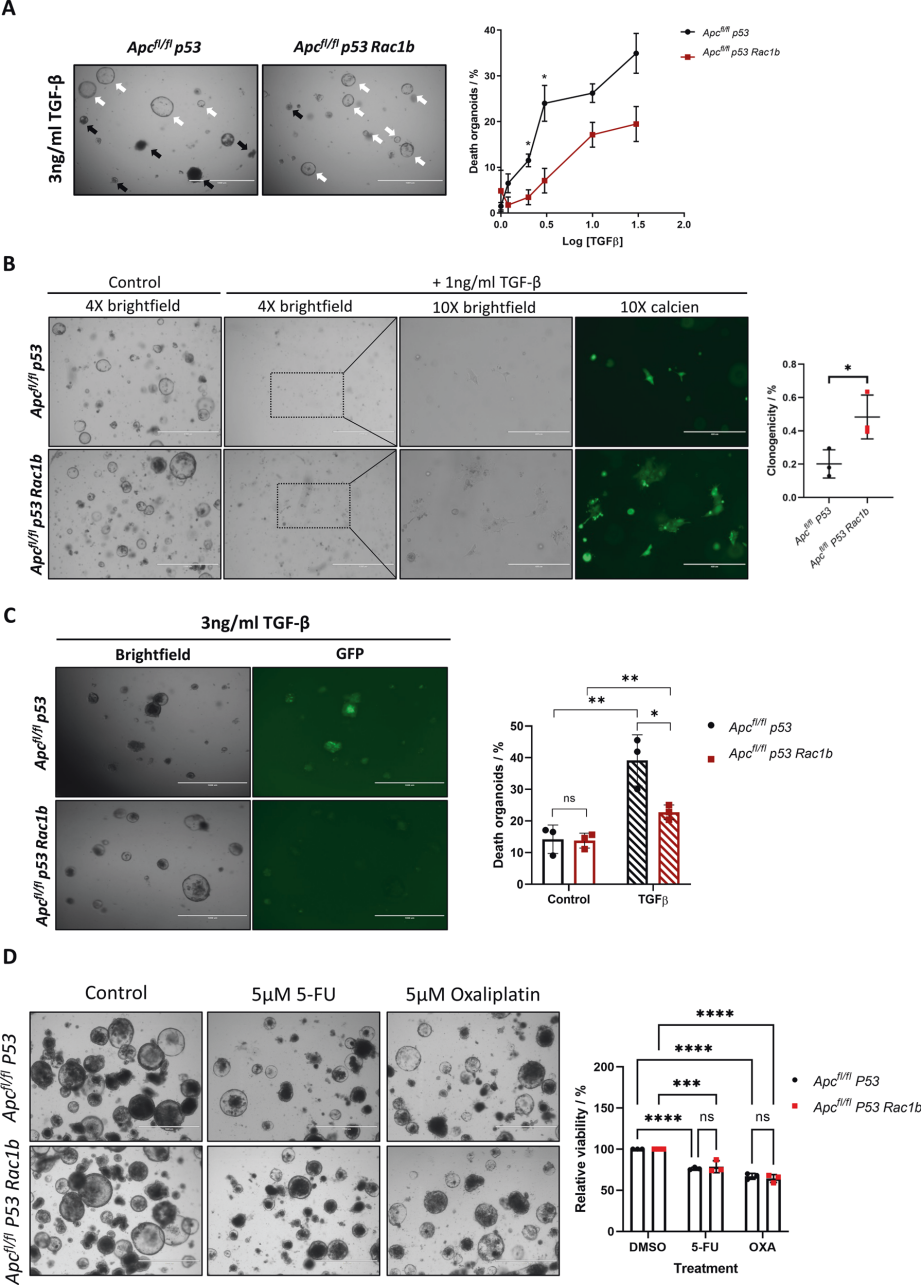
Overexpression of *RAC1B* suppresses TGFβ-induced cell death. **A** Representative images of *Apc*^*fl/fl*^ *p53* and *Apc*^*fl/fl*^ *p53 Rac1b* mouse-derived organoids treated with 3 ng/ml of TGFβ for 24 h. Quantification of organoids with a disrupted morphology (representative of organoid death) across different TGFβ concentrations is represented in the graph as percentage of disrupted organoids. Black and white arrows indicate disrupted (dead) and alive organoids, respectively. Scale bars are 1000 µm (error bars represent SEM; *P = 0.02; two tailed *t* test, *n* = 3vs3). **B** Clonogenicity assay of *Apc*^*fl/fl*^ *p53* and *Apc*^*fl/fl*^ *p53 Rac1b* organoids with or without 1 ng/ml of TGFβ for four days. Calcien staining is used to highlight surviving clones. Boxes indicate picture area magnified at 10X. Scale bars are 1000 µm (4X) and 400 µm (10X). Graph shows the percentage clonogenicity after four days in each cell line (error bars represent SD; *P = 0.0358; two tailed *t* test, *n* = 3vs3 independent biological replicates). **C** Apoptosis assay using the Caspase3/7 Green Detection Reagent of *Apc*^*fl/fl*^ *p53* and *Apc*^*fl/fl*^ *p53 Rac1b* organoids after treatment with 3 ng/ml of TGFβ for 24 h. Graph shows the percentage of cell death in organoids with or without TGFβ, scored as the area of green pixels relative to the total organoid area. Scale bars are 1000 µm (error bars represent SD; *P* ≥ 0.05, *P < 0.05, **P < 0.01; two tailed *t* test, *n* = 3vs3). **D** Representative images of *Apc*^*fl/fl*^ *p53* and *Apc*^*fl/fl*^ *p53 Rac1b* organoids treated with Oxaliplatin or 5-FU for 96 h (left panels). Resazurin viability assays of the same experiment to quantify cell viability (right panel) (error bars represent SD; ***P < 0.001, ****P < 0.0001; two way anova, *n* = 3vs3 independent biological replicates).

RAC1B has previously been shown to modulate TGFβ signalling [26] an inducer of apoptosis in early adenomatous cells [27]. We hypothesised that RAC1B-mediated suppression of TGFβ signalling may explain the reduced apoptotic rate in early adenomatous lesions in our model leading to increased rates of tumour initiation. To functionally assess the potential anti-apoptotic role of RAC1B following TGFβ stimulation we generated organoids from *VillinCreER*^*T2*^ *Apc*^*fl/fl*^ *TP53*^*fl/fl*^ mice with or without *RAC1B* overexpression (*Apc*^*fl/fl*^ *P53* or *Apc*^*fl/fl*^ *P53 Rac1b*) to model the early tumour-like lesions seen in the day-31 model. To investigate whether epithelial intestinal cells with *RAC1B* overexpression present a different response to TGFβ-induced cell death, we treated these organoids with increasing concentrations of TGFβ1 and monitored the appearance of organoids with disrupted morphology, indicative of organoid death [28]. Supporting our hypothesis, *RAC1B* overexpressing organoids treated with TGFβ presented a decreased proportion of dead organoids at all doses, showing significant differences at 1 ng/ml and 3 ng/ml (Fig. 4A). Likewise, organoids overexpressing *RAC1B* presented resistance to TGFβ1 driven cell death when cultured as single cells (Fig. 4B). To determine whether TGFβ1 induction of cell death was dependent on apoptosis, cell death was measured using Cell Event™ Caspase3/7 Green Detection Reagent following 24 h treatment with 3 ng/ml of TGFβ1. Upon Caspase-3 or -7 activation, a conjugated peptide is cleaved and releases a DNA binding dye, allowing the quantification of apoptotic death based on a fluorescent signal. While TGFβ1 significantly induced apoptosis in both cell lines (*p* = 0.0095 and *p* = 0.0094 in *Apc*^*fl/fl*^ *P53* and *Apc*^*fl/fl*^ *P53 Rac1b*, respectively), organoids bearing *RAC1B* overexpression were partially protected as they only presented an increase in the percentage of cell death of 8.95% compared to the 24.9% increase of the *Apc*^*fl/fl*^ *P53* organoids (*p* = 0.028, Fig. 4C). To determine whether reduced cell death was due to a general impairment of the apoptotic response we treated organoids with various agents known to induce apoptosis: the cytotoxic chemotherapeutic agents 5-FU and oxaliplatin. Organoids treated with 5-FU or oxaliplatin displayed significantly reduced viability compared to controls (Fig. 4D). However, there were no significant differences between genotypes, suggesting *RAC1B* overexpression is not imparting a general antiapoptotic effect (Fig. 4D). Altogether, these results suggest that overexpression of *RAC1B* confers protection to TGFβ1-driven apoptosis.

**Fig. 5:**
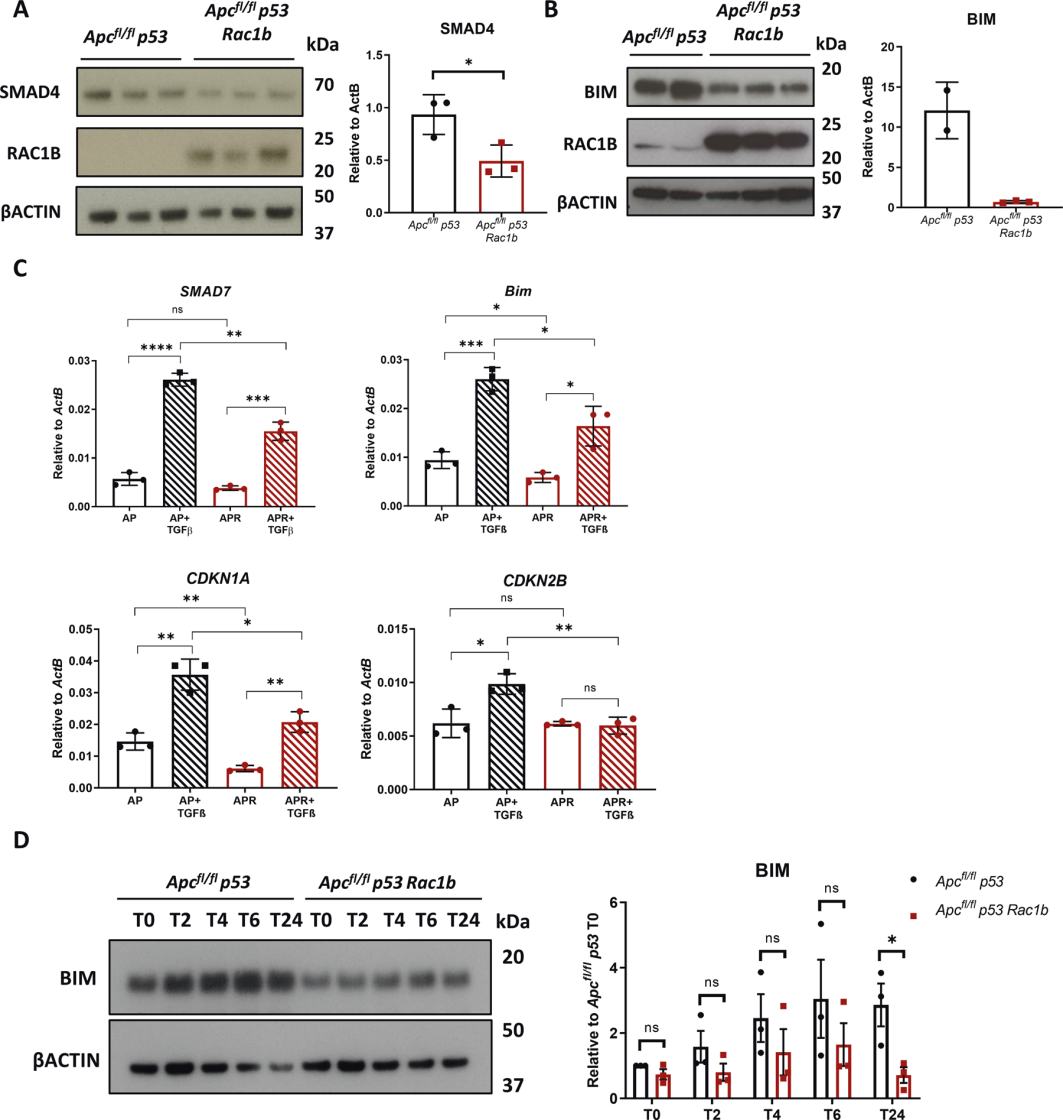
Overexpression of *RAC1B* impairs TGFβ pathway activation. **A** Western blot analysis of *Apc*^*fl/fl*^ *p53* and *Apc*^*fl/fl*^ *p53 Rac1b* organoids for SMAD4, RAC1B and βACTIN. Graph shows SMAD4 band densitometry quantification relative to βACTIN (error bars represent SD; *P = 0.034; two tailed *t* test, *n* = 3vs3). **B** Western blot analysis of *Apc*^*fl/fl*^ *p53* and *Apc*^*fl/fl*^ *p53 Rac1b* epithelial extractions for BIM, RAC1B and βACTIN. **C** qRT-PCR analysis of *SMAD7*, *BIM*, *CDKN1A* and *CDKN2B* of *Apc*^*fl/fl*^ *p53* and *Apc*^*fl/fl*^ *p53 Rac1b* organoids after 2 h treatment with 5 ng/ml TGFβ (error bars represent SD; n.s. *P* ≥ 0.05, *P < 0.05, **P < 0.01, ***P < 0.001, ****P < 0.0001; two tailed *t* test, *n* = 3vs3). **D** Western blot analysis for BIM and βACTIN of *Apc*^*fl/fl*^ *p53* and *Apc*^*fl/fl*^ *p53 Rac1b* organoids treated with 5 ng/ml TGFβ at baseline (T0), two (T2), four (T4), six (T6) and twenty-four (T24) hours. Graph represents BIM band densitometry quantification relative to βACTIN. Quantification is normalised to the expression of *Apc*^*fl/fl*^ *p53* at T0 (error bars represent SD; n.s. *P* ≥ 0.05, *P < 0.05; two tailed *t* test, *n* = 3vs3).

The TGFβ pathway is a superfamily of cytokine and receptors which, depending on the cellular context, can act as a tumour suppressor via cell growth inhibition and apoptosis or a tumour promoter via promotion of EMT and invasion [29, 30]. TGFβ signals through phosphorylation and nuclear translocation of the SMAD proteins, which act as transcription factors to initiate its gene expression program. One essential pathway transducer is SMAD4, which through binding to the phosphorylated receptor SMADs facilitate their translocation to the nucleus. Interestingly, SMAD4 levels in the *Apc*^*fl/fl*^ *P53 Rac1b* organoids were significantly reduced compared to *Apc*^*fl/fl*^ *P53* organoids, suggesting that the inability to fully respond to TGFβ1 might be due to intrinsic alterations in the pathway (Fig. 5A). They also express lower levels of BIM, a pro-apoptotic protein and key mediator of TGFβ-induced cell death [31] (Fig. 5B). To investigate whether *RAC1B* overexpression alters the response to TGFβ1, we treated *Apc*^*fl/fl*^ *P53* and *Apc*^*fl/fl*^ *P53 Rac1b* organoids with TGFβ1 for 2 h and checked the transcriptional expression of TGFβ1 target genes. Whilst both groups responded to TGFβ1 by significantly upregulating the expression of these genes, organoids with *RAC1B* overexpression showed limited response either because of reduced expression at basal levels (*Bim* and *Cdkn1a*), because of a lower induction (*Smad7*) or because of an inability to respond at all (*Cdkn2b*) (Fig. 5C). Given the resistance to TGFβ1-driven cell death observed in organoids overexpressing *RAC1B*, the regulation of *Bim* expression was of particular interest. To explore this further, we conducted a time-course induction with TGFβ1 and assessed the induction of BIM at protein level by Western blot. Interestingly, *Apc p53 Rac1b* organoids failed to fully induce BIM protein expression across all time-points, never reaching the expression of control organoids (Fig. 5D). Together, these data indicate that RAC1B attenuates the activation of the TGFβ signalling pathway, protecting against the induction of cell death.

### High levels of RAC1B correlate with a reduced activation of TGFβ in vivo

**Fig. 6:**
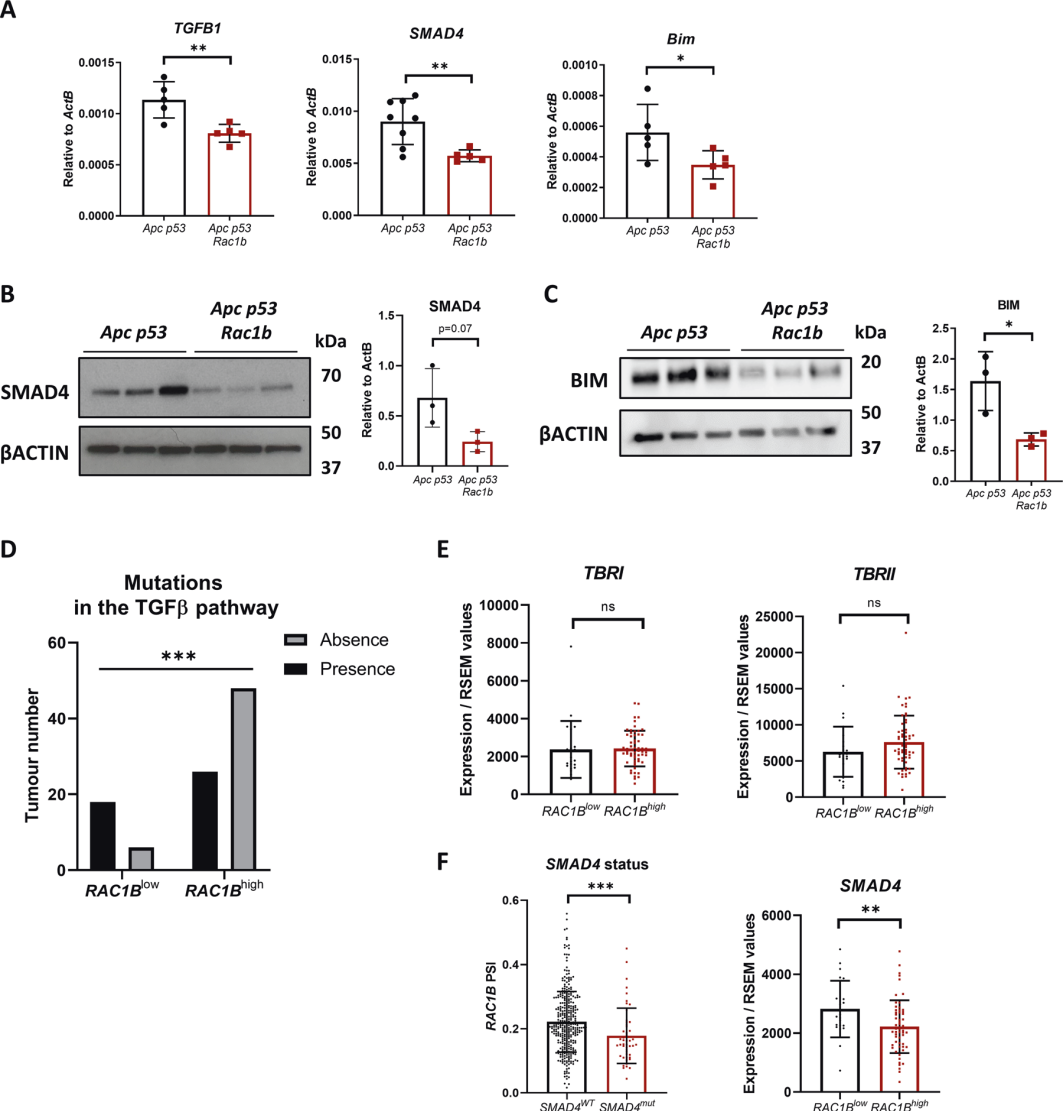
Negative regulation of TGFβ signalling by RAC1B in vivo. **A** qRT-PCR analysis of *TGFB1*, *SMAD4* and *BIM* gene expression of SI from *Apc p53* and *Apc p53 Rac1b* mice after 31 days of tamoxifen induction for gene expression (error bars represent SD; *P < 0.05, **P < 0.01; two tailed *t* test, *n* = 5vs5). **B** Western blot analysis of day-31 *Apc p53* and *Apc p53 Rac1b* intestinal epithelial extractions protein lysates for SMAD4 and βACTIN. Graph represents SMAD4 band densitometry quantification relative to βACTIN (error bars represent SD; #*P* < 0.10; two tailed *t* test, *n* = 3vs3). **C** Western blot analysis of day-31 *Apc p53* and *Apc p53 Rac1b* intestinal epithelial extractions protein lysates for BIM and βACTIN. Graph represents BIM band densitometry quantification relative to βACTIN (error bars represent SD; *P < 0.05; two tailed *t* test, *n* = 3vs3). **D** Correlation analysis of COAD tumours from the TCGA database analysing *RAC1B*^*low*^ and *RAC1B*^*high*^ tumours based on the presence or absence of mutations in a defined set of genes involved in the TGFβ pathway (***P < 0.001, two-sided Fisher’s test, *n* = 24vs74). **E** Expression of TGFβ receptor 1 (*TBRI*) and TGFβ receptor 2 (*TBRII*) in *RAC1B*^*low*^ and *RAC1B*^*high*^ tumours in the COAD TCGA database (error bars represent SD; *P* ≥ 0.10; two tailed *t* test, *n* = 22vs59). **F** On the left, graph presenting COAD tumours with (*SMAD4*^*mut*^) or without (*SMAD4*^*wt*^) mutations in the *SMAD4* gene base on the percentage spliced-in (PSI) of *RAC1B*. On the right, expression of *SMAD4* in *RAC1B*^*low*^ and *RAC1B*^*high*^ tumours in the COAD TCGA database (error bars represent SD; **P < 0.01; two tailed *t* test, *n* = 22vs59).

Early adenomas arising in the day-31 pre-tumorigenic model with *RAC1B* overexpression present a reduced number of apoptotic cells (Fig. 3E). To investigate whether these mice also bear alterations in the TGFβ signalling pathway, we looked at the expression of *TGFβ1*, *SMAD4* and *Bim* by qRT-PCR in SI tissue. Interestingly, mice from the *Apc p53 Rac1b* group had reduced expression of those genes (Fig. 6A). We also investigated the expression of SMAD4 and BIM protein, which showed the same results (Fig. 6B, C). Overall, this indicates that RAC1B regulates members of the TGFβ pathway in vivo, potentially leading to reduced activity of the pathway, favouring the development of transformed cells into adenomas.

Given its tumour suppressor role, inactivating mutations in the TGFβ signalling pathway is a common tumourigenic mechanism in CRC. Taking advantage of our previous strategy to analyse TCGA tumour data based on *RAC1B* percentage spliced-in (PSI) [17], we looked at the association between tumours with high or low *RAC1B* expression (*RAC1B*^*high*^ or *RAC1B*^*low*^) and the presence or absence of mutations in genes involved in the TGFβ pathway. These genes constitute a list of 43 core members of the canonical TGFβ pathway including TGFβ ligands, bone morphogenic protein (BMP) and activin (ACV) ligands and receptors, among others [32] (Supplementary Table 1). Analysis of the colorectal cancer (COAD) dataset demonstrated that tumours with high *RAC1B* expression are less prone to present mutations within the pathway, suggesting that high levels of *RAC1B* may be an alternative, non-mutational mechanism for reducing pathway activity (*p* = 0.0009, Fig. 6D).

Previous data in the literature has suggested that RAC1B might interfere with the TGFβ signalling pathway by reducing the expression of the TGFβ receptor I (TβRI/ALK5) [33]. To investigate whether human tumours present this negative association, we looked at the expression of *TβRI* as well as of *TβRII* based on tumours with high or low *RAC1B* PSI from the COAD dataset. However, no significant differences were observed between groups, suggesting that RAC1B may regulate the TGFβ signalling through mechanisms other than modifying the expression of the receptors (Fig. 6E).

**Fig. 7:**
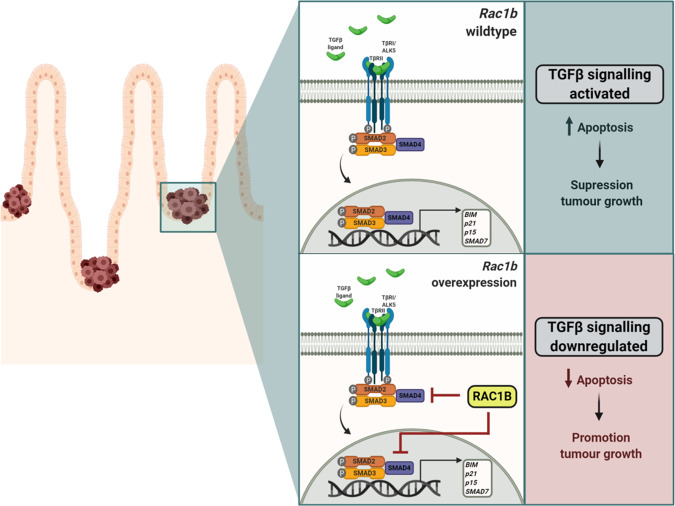
Model of RAC1B negative regulation of TGFβ signalling promoting tumour initiation. Schematic outlining of RAC1B function in our model: when pre-adenomatous lesions are formed in the small intestine under low/wild type levels of *RAC1B* expression, TGFβ remains active and can induce apoptosis through transcription activation of its targeted genes such as *BIM*, *CDKN1A* and *CDKN2B*, leading to the suppression of tumour growth and favouring survival. However, in the context of high *RAC1B* levels, TGFβ signalling activation is impaired, resulting in a downregulation of its activity and a protection of its tumour suppressive effect, thereby promoting tumour growth and worsening survival. Figure created with BioRender.com.

*SMAD4* is the most commonly mutated gene of the TGFβ pathway in CRC, with a mutational frequency of around 13% [32]. Bearing in mind the negative regulation of SMAD4 that we have observed in organoids and mice with *RAC1B* overexpression (Figs. 5A and 6A, B), we investigated whether there is an association between *RAC1B* expression and *SMAD4* mutational status. Interestingly, tumours with wild-type *SMAD4* express significantly more *RAC1B*, indicating high *RAC1B* expression might compensate for its lack of mutation (*p* = 0.0004, Fig. 6F). Likewise, *SMAD4* expression is significantly lower in the tumours within the *RAC1B*^*high*^ group (*p* = 0.0063, Fig. 6F), suggesting a negative association between *SMAD4* mutation and *RAC1B* expression. Moreover, analysis of the apoptotic genes *CASPASE-3* and *-7* on the TCGA dataset revealed that *RAC1B*^*high*^ tumours express significantly lower levels, indicating that *RAC1B* might promote apoptosis resistance in human CRC (Fig. S3A). Together, our data suggest that RAC1B constitutes a mechanism, both in mouse CRC models and human CRC tumours, to downregulate TGFβ signalling and promote tumorigenesis (Fig. 7).

## Discussion

Since its discovery 20 years ago, the main oncogenic role assigned to RAC1B in the literature has been a driver of tumour invasion, yet functional in vivo evidence is lacking. Here, we aimed to characterise the invasive role of RAC1B utilising a mouse model of colorectal cancer with potential invasive properties. Mice carried a conditional co-deletion of the *Apc* and *TP53* alleles and overexpression of a human *RAC1B* cDNA. However, neither of the genotypes (*Apc p53* nor *Apc p53 Rac1b*) developed invasive tumours and *RAC1B* overexpressing mice were terminated early due to enhanced tumour burden. When modelling tumour invasion, time is a limiting factor and mice would ideally present a low tumorigenic rate, creating a permissive environment for tumours developing as aggressive carcinomas. This could indicate that the speed of our model is not suitable to allow invasion. Therefore, alternative models are needed for investigating RAC1B invasive-related roles. In spite of this, we observed a striking tumour initiation phenotype driven by RAC1B, as *RAC1B* overexpression increased tumour number and reduced survival. As endpoint tumours did not apparently manifest gross differences, a pre-tumorigenic model was utilised to study early cellular transformation events driven by RAC1B. Intriguingly, a number of microlesions were detected 31 days after tamoxifen induction, which were characterised as early adenomas given their nuclear localisation of β-catenin. Given the requirement for *Rac1b* to fully activate Wnt signalling in CRC [17, 24], the most feasible explanation would be over activation of Wnt activity. However, we did not observe increased expression of Wnt target genes in tumours, microlesions or normal tissue. A possible explanation is that in the context of *Apc* and *TP53* loss, activation of Wnt signalling is no longer sensitive to overexpression of *RAC1B*. A number of previous studies have demonstrated that loss of *TP53* can activate Wnt signalling in various contexts, supportive of this possibility [34, 35]. Instead, we observed significantly less apoptotic cells in tumour microlesions overexpressing *RAC1B*, suggesting RAC1B might favour tumourigenesis by protecting from cell death.

Functional investigation showed that *RAC1B* conferred specific protection against TGFβ-induced apoptosis through downregulation of its transcriptional activity. Moreover, human data from the COAD TCGA database revealed that (i) tumours with high *RAC1B* express lower levels of the apoptotic genes Caspase-3 and -7, (ii) higher expression of *RAC1B* correlates with fewer mutations within the TGFβ signalling pathway and (iii) a strong negative association exists between *SMAD4* mutations and *RAC1B* expression. Together, this suggests that tumours expressing high *RAC1B* levels might suppress TGFβ signalling through means other than acquired mutations in the pathway.

An association between RAC1B and the TGFβ pathway has been suggested previously [26]. Similar to our findings, RAC1B was found to inhibit TGFβ by a number of mechanisms, including downregulation of SMAD4 [36]. Their work focused on the tumour promoter role of TGFβ, finding that its RAC1B-dependent inhibition leads to a suppression of cell proliferation and migration in different PAAD and malignant breast cell lines [37]. TGFβ is a multimodal signalling pathway, which affects numerous downstream cues, making the outcome of its inhibition highly dependent on the cellular context [38]. The different experimental settings in which the TGFβ and RAC1B interaction has been studied likely explain the different conclusions from our study. Therefore, it appears that suppression of TGFβ signalling is a key downstream effect of RAC1B signalling and whether this leads to tumour promoting or suppressing effects is dependent on the context of this overexpression. In the context of colorectal cancer, the building evidence from the literature [14, 19–22, 24] along with our in vivo mouse experiments and the analysis of human cancer expression data [32] suggests that RAC1B acts as a tumour promoting factor.

Another aspect that remains uncovered is the mechanism through which RAC1B modulates TGFβ and BIM downregulation. Previous data has suggested that RAC1B inhibits TGFβ by interfering at different levels of the pathway, including inhibition of TGFBR1/ALK5 expression [39], reduction of SMAD2/3 phosphorylation [39], downregulation of SMAD4 [36] and induction of USP26, which stabilises SMAD7 inhibiting TGFβ [40]. We have observed SMAD4 downregulation in organoids and tissue overexpressing *RAC1B*, and COAD TCGA data points towards a link between SMAD4 and RAC1B. However, whether RAC1B modulates TGFβ activity directly through downregulation of SMAD4 warrants further investigation.

Collectively, we have described a link between RAC1B and the TGFβ pathway in the context of colorectal cancer, whose modulation favours tumour promotion through an anti-apoptotic effect. This work positions RAC1B as a complex player, orchestrating different cellular changes and signalling cascades with important pro-tumourigenic functions.

## Material and methods

### Mouse experiments

All experiments were performed in compliance with UK Home Office regulations. Mice were bred at the animal facilities of the University of Edinburgh and the Beatson Institute. Mice were maintained in 12 h light–dark cycles and were given access to food and water at libitum. Mice colonies of both sexes with a mixed background were used throughout. Mice were genotyped by Transnetyx (Cordoba, USA). All mice carried a tamoxifen inducible Cre recombinase gene under control of the Villin promoter. The alleles used for this study were as follows: *Vil-Cre-ER*^*T2*^ [41], *Apc* [42], 580S, *TP53*^*fl*^ [43]. Overexpression of *RAC1B* was achieved by knocking in a human *RAC1B* cDNA into the Rosa26-lox-Stop-lox locus [23]. Between 6 and 12 weeks of age, or when they reached a minimum weight of 20 g, mice were induced with tamoxifen by an intraperitoneal (IP) injection. For the tumour cohort, mice were induced with a single dose of 80 mg/kg of tamoxifen and were aged until they reached end point (pale feet, weight loss, rectal bleeding and/or hunched position). For the day-31 cohort, mice were sacrificed after 31 days of tamoxifen injection. Mice were monitored and assessed for symptoms at least twice a week. For short-term Cre recombination, mice were induced by 120 mg/kg and 80 mg/kg of tamoxifen at day 1 and day 2, respectively, and were sacrificed at day 5 post induction. Mice were sacrificed by cervical dislocation. To assess proliferation of the intestinal epithelium, mice were injected with a single IP injection of 200 µl of BrdU cell proliferation label (GE, 1392253) 2 h prior sacrifice. Tumour number and tumour burden of long-term cohort was scored macroscopically in situ after mouse sacrifice. Tumour number of day-31 cohort was quantified microscopically by H&E serial sections of the small intestine.

Sample sizes for each experiment are outlined in the figure legends. For all animal experiments, *n* = 3 or *n* > 3 mice were used for each experimental cohort. Power analyses were carried out prior to experiments being carried out to determine the minimum number of animals required for each experiment. These analyses were informed by previous and / or preliminary experiments. No animals were excluded from downstream analysis. Animals were not randomised and were assigned to different groups based on genotype. Investigators were blinded to the genotype of the animals during husbandry, clinical assessment and dissection. Investigators were blinded to animal genotype during sample analysis.

### Crypt isolation

Small intestine was harvested, flushed with cold PBS, opened longitudinally and scrapped off with a cover slip to discard villi. Tissue was cut into 5 mm pieces, transferred to a 50 ml falcon tube and washed with 10 ml of cold PBS up to five times. Tissue was digested with 2 mM EDTA for 30 min at 4 °C with agitation. EDTA was washed off with PBS and tissue was thoroughly pipetted up and down about ten times to loosen intestinal cells. Supernatant was collected and this step was repeated three more times. Supernatants were centrifuged at 300 *g* for 10 min at 4 °C. Pellet corresponds to intestinal epithelial extractions and these were used for organoid culture, RNA extraction and/or epithelial extractions.

### Organoid culture

Crypts were isolated as previously described but only the last two washes (the most crypt-enriched supernatants) were collected and centrifuged. Pellet was resuspended with 10 ml of Advanced DMEM/F-12 medium (ADF, Gibco) supplemented with 100U/ml penicillin, 100 µg/ml streptomycin, 2mM L-Glutamine and 10 mM HEPES (Life technologies (15630080) (from now on, medium will be referred as ADF) and passed through a 70 µm cell strainer. Crypts were spun at 100 *g* for 10 min. Pellet was then carefully resuspended in an appropriate volume of Matrigel (Corning) and a volume of 10 µl per well was plated in a pre-warmed 24-well plate. Plate was incubated at 37 °C for at least 10 min to allow Matrigel solidification. A volume of 500 µl/well of growth media was added, composed of ADF, 1X B27 (Gibco, 17504044), 1X N2 (Gibco, (17502048), 100 ng/ml Noggin (Peprotech, 250-38-500) and 50 ng/ml EGF (Peprotech, 315-09-500). For tumour organoid culture, tumours were cut into small pieces and washed with ADF. After washing, tumours were digested in 2 ml of digestion media, composed of: 1700µl ADF, 1 mg/ml Collagenase II (Sigma, C1764), 0.5 mg/ml Hyalurodinase (Sigma, H3606) and 10 µM Y27632 (Tocris, 1254). Digestion was conducted for 90 min at 37 °C and vigorous pipetting was done every 15 min to enhance digestion. Digestion was stopped with 1% BSA, cells were filtered through a 40 µm cell strainer and cells were centrifuged at 100 *g* for 3 min. Pellet was resuspended in 500 µl of Matrigel and plating was conducted as with isolated crypts. After 3 to 4 days, organoids were passaged. Organoids were cultures from mice of the following genotypes *VilCre*^*ERT2*^ *Apc*^*fl/fl*^ *TP53*^*fl/fl*^ and *VilCre*^*ERT2*^ *Apc*^*fl/fl*^ *TP53*^*fl/fl*^ *Rosa*^*lsl-hRAC1B/lsl-hRAC1B*^. Organoid cultures were routinely tested for Mycoplasma contamination.

### Clonogenicity assays

Cultured crypts were dissociated from the Matrigel and washed twice with cold PBS at 300 *g* for 5 min. Crypts were then digested into single cells with StemPro Accutase solution (Life Technologies, A1110501) for 10 min at 37 °C. Digestion was stopped with ADF and cells were filtered through a 40 µm cell strainer. Cells were spun down at 100 *g* for 5 min and counted using an automated cell counter (Countess II, Thermo Fisher, A27974). One thousand single cells per 5 µl/drop of Matrigel were plated in a 24-well plate and a minimum of five drops per genotype was plated per each technical replicate. Each experiment was conducted with three biological replicates per genotype. After Matrigel solidification, growth media was added as usual and formed spheroid clones were scored after 4 days. Clonogenicity assays were also carried out in the presence of 1 ng/ml TGFβ1 (Peprotech, 100-21-10). In these experiments, clones spread in response to TGFβ1 treatment and calcien was used to highlight them for identification and scoring.

### Organoid cell death experiments

Organoids were dissociated, washed and digested as fragments (small cell aggregates made up of approximately 10 single cells). Crypt fragments were achieved by mechanical disaggregation, resuspending the cell pellet gently with a p1000 for 30–40 times. Forty fragments per 4 µl/drop of Matrigel were plated and clones were scored 48 h later. At that time, growth media was replaced with fresh media supplemented TGFβ1 (Peprotech, 100-21-10). The following titration of doses was used: 0, 0.3 ng/ml, 1 ng/ml, 3 ng/ml, 10 ng/ml and 30 ng/ml. After 24 h of treatment, number of organoids with a dead morphology was scored [28]. At least eight wells per each cell line were plated, and three biological replicates per each genotype were used in every experiment. Percentage of cell death was calculated based on the number of dead organoids relative to the initial number of clones formed.

To measure the number of apoptotic cells, the CellEvent Caspase-3/7 Green Detection Reagent (Invitrogen, C10423) was added to the TGFβ-supplemented media. After 24 h of treatment apoptosis was measured with ImageJ on Brightfield and fluorescent images. Brightfield images were used as a reference for organoid region of interest (ROIs). Fluorescent images were overlaid and the percentage of green pixels per ROI was calculated. This assay was validated in parallel with simultaneous scoring of cell death as described above.

For oxaliplatin and 5-FU treatment experiments, organoids were mechanically dissociated into small fragments and seeded in 5ul Matrigel in a 24-well cell culture plate. 24 h after seeding, medium was replaced with fresh medium containing 5 μM oxaliplatin or 5 μM 5-FU or DMSO. Resazurin assays were carried out 96 h later to determine relative cell viability.

### Acute and time-course treatment with TGFβ

Organoids were digested as fragments (small cell aggregates made up of approximately 10 single cells) and plated at a confluency of 100 fragments/µl of Matrigel. Three days after, media was replaced with growth media supplemented with or without 5 ng/ml of TGFβ1. Organoids were collected 2 h after and RNA was isolated. For time-course expression analysis of BIM, organoids were collected after 2, 4, 6 and 24 h of treatment and protein was isolated.

### Statistical analysis

GraphPad Prism v7.0 software (La Jolla) was used for all statistical analysis. Comparison of two groups was assessed by Student’s *t*-test, two-tailed, parametric and unpaired if groups followed normality. Otherwise, non-parametric Mann–Whitney test was used for groups that did not follow normality. Percentage of survival was calculated by a Log-rank (Mantel–Cox) test. Association between two categories was assessed by two-sided Fisher’s test. *P* values lower than 0.05 are considered statistically significant. Non-significant values (*p* ≥ 0.05) are labelled as n.s. For all figures, the statistical test are justified as appropriate and the data meet the assumptions of the tests. Estimates of variation are included for each group of data (SD or SEM) and are reported in the corresponding figure legends. The variance is similar between groups that are being statistically compared.

## Supplementary information

Supplemental Material

## Data Availability

Requests for further information, reagents and resources should be directed to and will be fulfilled by the Lead Contact, Kevin B. Myant: (kevin.myant@igmm.ed.ac.uk).

## References

[1] Bray F, Ferlay J, Soerjomataram I, Siegel RL, Torre LA, Jemal A (2018). Global cancer statistics 2018: GLOBOCAN estimates of incidence and mortality worldwide for 36 cancers in 185 countries. CA Cancer J Clin.

[2] Sanchez-Vega F, Mina M, Armenia J, Chatila WK, Luna A, La KC (2018). Oncogenic signaling pathways in The Cancer Genome Atlas. Cell.

[3] Kandoth C, McLellan MD, Vandin F, Ye K, Niu B, Lu C (2013). Mutational landscape and significance across 12 major cancer types. Nature.

[4] Fearon ER, Vogelstein B (1990). A genetic model for colorectal tumorigenesis. Cell.

[5] Nakayama M, Sakai E, Echizen K, Yamada Y, Oshima H, Han T-S (2019). Intestinal cancer progression by mutant p53 through the acquisition of invasiveness associated with complex glandular formation. Oncogene.

[6] Schwitalla S, Ziegler PK, Horst D, Becker V, Kerle I, Begus-Nahrmann Y (2013). Loss of p53 in enterocytes generates an inflammatory microenvironment enabling invasion and lymph node metastasis of carcinogen-induced colorectal tumors. Cancer Cell.

[7] Colicelli J (2004). Human RAS superfamily proteins and related GTPases. Sci STKE.

[8] Hodge RG, Ridley AJ (2016). Regulating Rho GTPases and their regulators. Nat Rev Mol Cell Biol.

[9] Matos P, Skaug J, Marques B, Beck S, Veríssimo F, Gespach C (2000). Small GTPase Rac1: structure, localization, and expression of the human gene. Biochem Biophys Res Commun.

[10] Fiegen D, Haeusler L-C, Blumenstein L, Herbrand U, Dvorsky R, Vetter IR (2004). Alternative splicing of Rac1 generates Rac1b, a self-activating GTPase. J Biol Chem.

[11] Kazanietz MG, Caloca MJ (2017). The Rac GTPase in cancer: from old concepts to new paradigms. Cancer Res.

[12] Myant KB, Cammareri P, McGhee EJ, Ridgway RA, Huels DJ, Cordero JB (2013). ROS production and NF-κB activation triggered by RAC1 facilitate WNT-driven intestinal stem cell proliferation and colorectal cancer initiation. Cell Stem Cell.

[13] Pickering KA, Gilroy K, Cassidy JW, Fey SK, Najumudeen AK, Zeiger LB (2021). A RAC-GEF network critical for early intestinal tumourigenesis. Nat Commun.

[14] Jordan P, BrazÃo R, Boavida MG, Gespach C, Chastre E (1999). Cloning of a novel human Rac1b splice variant with increased expression in colorectal tumors. Oncogene.

[15] Schnelzer A, Prechtel D, Knaus U, Dehne K, Gerhard M, Graeff H (2000). Rac1 in human breast cancer: overexpression, mutation analysis, and characterization of a new isoform, Rac1b. Oncogene.

[16] Ryan M, Wong WC, Brown R, Akbani R, Su X, Broom B (2016). TCGASpliceSeq a compendium of alternative mRNA splicing in cancer. Nucleic Acids Res.

[17] Gudiño V, Pohl SÖG, Billard CV, Cammareri P, Bolado A, Aitken S (2021). RAC1B modulates intestinal tumourigenesis via modulation of WNT and EGFR signalling pathways. Nat Commun.

[18] Radisky DC, Levy DD, Littlepage LE, Liu H, Nelson CM, Fata JE (2005). Rac1b and reactive oxygen species mediate MMP-3-induced EMT and genomic instability. Nature.

[19] Pelisch F, Khauv D, Risso G, Stallings-Mann M, Blaustein M, Quadrana L (2012). Involvement of hnRNP A1 in the matrix metalloprotease-3-dependent regulation of Rac1 pre-mRNA splicing. J Cell Biochem.

[20] Stallings-Mann ML, Waldmann J, Zhang Y, Miller E, Gauthier ML, Visscher DW (2012). Matrix metalloproteinase induction of Rac1b, a key effector of lung cancer progression. Sci Transl Med.

[21] Esufali S, Charames GS, Pethe VV, Buongiorno P, Bapat B (2007). Activation of tumor-specific splice variant Rac1b by dishevelled promotes canonical Wnt signaling and decreased adhesion of colorectal cancer cells. Cancer Res.

[22] Dai B, Zhang X, Shang R, Wang J, Yang X, Zhang H (2018). Blockade of ARHGAP11A reverses malignant progress via inactivating Rac1B in hepatocellular carcinoma. Cell Commun Signal.

[23] Zhou C, Licciulli S, Avila JL, Cho M, Troutman S, Jiang P (2013). The Rac1 splice form Rac1b promotes K-ras-induced lung tumorigenesis. Oncogene.

[24] Kotelevets L, Walker F, Mamadou G, Lehy T, Jordan P, Chastre E (2018). The Rac1 splice form Rac1b favors mouse colonic mucosa regeneration and contributes to intestinal cancer progression. Oncogene.

[25] Jackstadt R, van Hooff SR, Leach JD, Cortes-Lavaud X, Lohuis JO, Ridgway RA (2019). Epithelial NOTCH signaling rewires the tumor microenvironment of colorectal cancer to drive poor-prognosis subtypes and metastasis. Cancer Cell.

[26] Ungefroren H, Sebens S, Giehl K, Helm O, Groth S, Faendrich F (2014). Rac1b negatively regulates TGF-β-induced cell motility in pancreatic ductal epithelial cells by suppressing Smad signalling. Oncotarget.

[27] Wiener Z, Band AM, Kallio P, Högström J, Hyvönen V, Kaijalainen S (2014). Oncogenic mutations in intestinal adenomas regulate Bim-mediated apoptosis induced by TGF-β. Proc Natl Acad Sci USA.

[28] Grabinger T, Luks L, Kostadinova F, Zimberlin C, Medema JP, Leist M (2014). Ex vivo culture of intestinal crypt organoids as a model system for assessing cell death induction in intestinal epithelial cells and enteropathy. Cell Death Dis.

[29] Batlle E, Massagué J (2019). Transforming growth factor-β signaling in immunity and cancer. Immunity.

[30] Jung B, Staudacher JJ, Beauchamp D (2017). Transforming growth factor β superfamily signaling in development of colorectal cancer. Gastroenterology.

[31] Ramesh S, Wildey GM, Howe PH (2009). Transforming growth factor β (TGFβ)-induced apoptosis: the rise and fall of Bim. Cell Cycle.

[32] cBioPortal for Cancer Genomics. Available from: https://www.cbioportal.org/ (Accessed: 27^th^ May 2020).

[33] Witte D, Otterbein H, Förster M, Giehl K, Zeiser R, Lehnert H (2017). Negative regulation of TGF-β1-induced MKK6-p38 and MEK-ERK signalling and epithelial-mesenchymal transition by Rac1b. Sci Rep.

[34] Wellenstein MD, Coffelt SB, Duits DEM, van Miltenburg MH, Slagter M, de Rink I (2019). Loss of p53 triggers WNT-dependent systemic inflammation to drive breast cancer metastasis. Nature.

[35] Kim NH, Kim HS, Kim NG, Lee I, Choi HS, Li XY (2011). p53 and microRNA-34 are suppressors of canonical Wnt signaling. Sci Signal.

[36] Ungefroren H, Wellner UF, Keck T, Lehnert H, Marquardt JU (2020). The small gtpase rac1b: a potent negative regulator of-and useful tool to study-tgfβ signaling. Cancers.

[37] Melzer C, Hass R, Von Der Ohe J, Lehnert H, Ungefroren H (2017). The role of TGF-β and its crosstalk with RAC1/RAC1b signaling in breast and pancreas carcinoma. Cell Commun Signal.

[38] David CJ, Massagué J (2018). Contextual determinants of TGFβ action in development, immunity and cancer. Nat Rev Mol Cell Biol.

[39] Ungefroren H, Otterbein H, Fiedler C, Mihara K, Hollenberg MD, Gieseler F (2019). RAC1B suppresses TGF-β1-dependent cell migration in pancreatic carcinoma cells through inhibition of the TGF-β Type I receptor ALK5. Cancers.

[40] Ungefroren H, Kumarasinghe A, Musfeldt M, Fiedler C, Lehnert H, Marquardt JU (2020). RAC1B induces SMAD7 via USP26 to suppress TGFβ1-dependent cell migration in mesenchymal-subtype carcinoma cells. Cancers.

[41] El Marjou F, Janssen K-P, Hung-Junn Chang B, Li M, Hindie V, Chan L (2004). Tissue-specific and inducible Cre-mediated recombination in the gut epithelium. Genesis.

[42] Shibata H, Toyama K, Shioya H, Ito M, Hirota M, Hasegawa S (1997). Rapid colorectal adenoma formation initiated by conditional targeting of the Apc gene. Science.

[43] Jonkers J, Meuwissen R, van der Gulden H, Peterse H, van der Valk M, Berns A (2001). Synergistic tumor suppressor activity of BRCA2 and p53 in a conditional mouse model for breast cancer. Nat Genet.

