# Supplementary material for: Negative regulation of TGFβ-induced apoptosis by RAC1B enhances intestinal tumourigenesis
Source: Cell Death Dis. 2021 Sep 25;12(10):873. doi: 10.1038/s41419-021-04177-7 (PMC8464603; doi:10.1038/s41419-021-04177-7)
Supplement: Supplementary file 1 — Supplemental Material [file 41419_2021_4177_MOESM1_ESM.docx]

**Gudiño et al., Supplementary Data**

Figure S1

Figure S2

Figure S3

Supplementary Table 1

Supplementary Materials and Methods


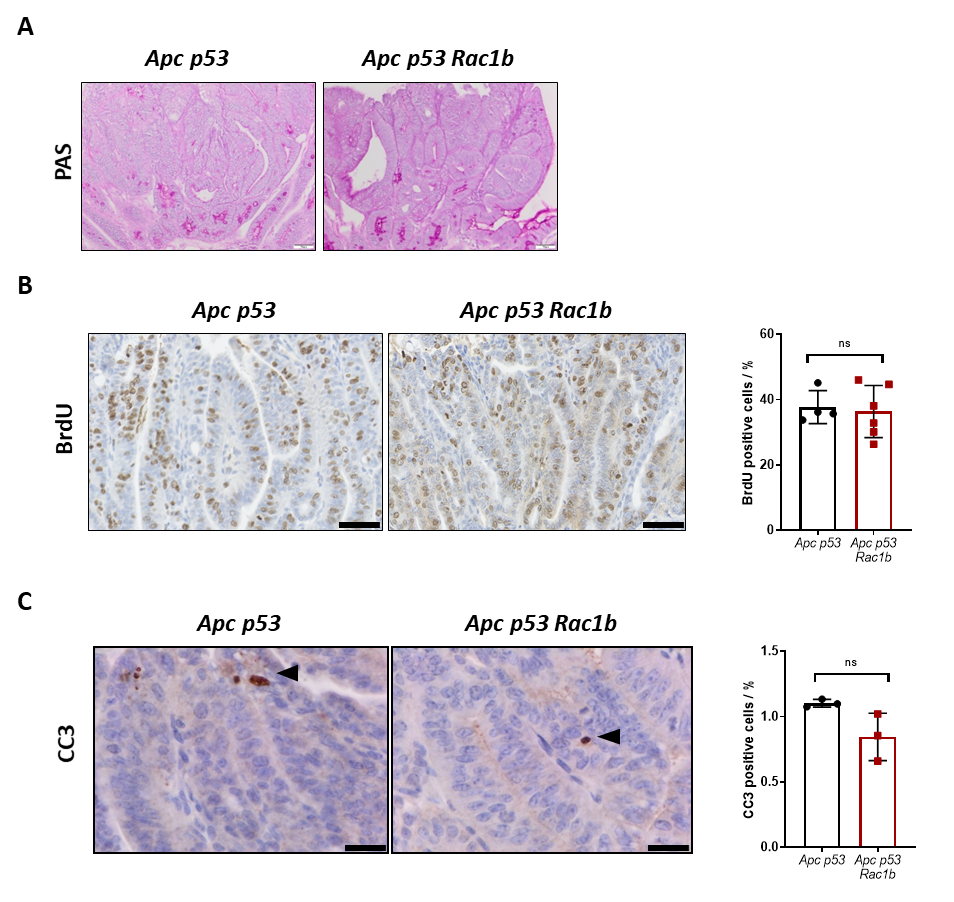


**Figure S1. Overexpression of *RAC1B* promotes tumourigenesis and decreases mouse survival.**. (A) Periodic acid-Schiff (PAS) staining in small intestine tumours of *Apc p53* and *Apc p53 Rac1b* mice. Scale bars are 50µm. (B) Representative images of BrdU IHC in tumours from *Apc p53* and *Apc p53 Rac1b* mice. Percentage of proliferation is shown based on the number of BrdU positive cells relative to the total number of tumour cells. Scale bars are 100µm (error bars represent SD; P≥0.05; two tailed t test, n=4vs6). (C) Representative images of CC3 IHC in tumours from *Apc p53* and *Apc p53 Rac1b* mice. Percentage of apoptosis is shown based on the number of CC3 positive cells relative to the total number of tumour cells. Scale bars are 20µm (error bars represent SD; #P<0.1; two tailed t test, n=3vs3).


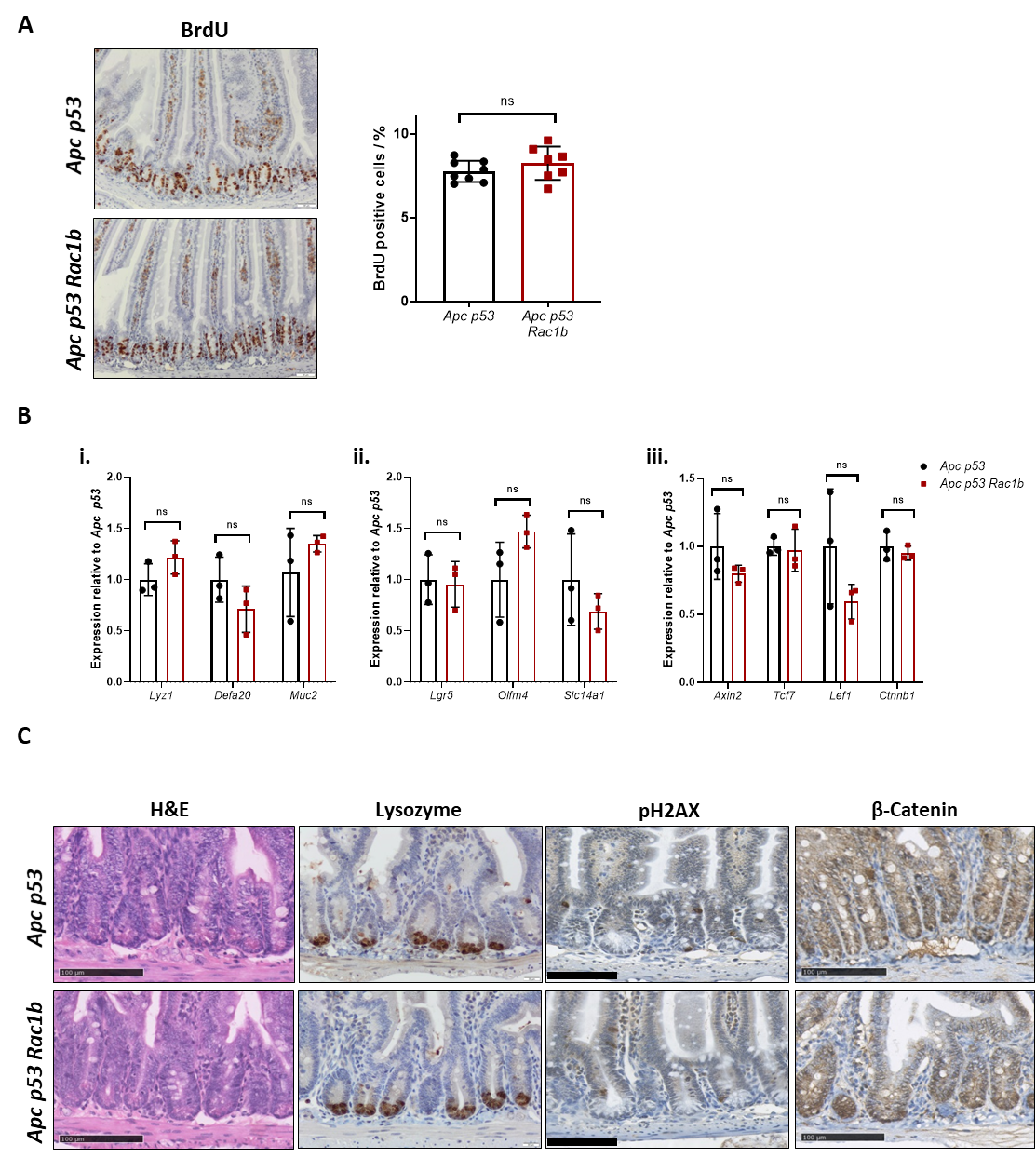


**Figure S2. Overexpression of *RAC1B* does not alter intestinal homeostasis in the normal tissue.** (A) BrdU IHC of small intestine tissue from *Apc p53* and *Apc p53 Rac1b* mice after 31 days of tamoxifen induction. Percentage of proliferation is calculated as the average number of crypt BrdU+ cells per mouse. Thirty crypts were scored per mouse. Scale bars are 50µm (error bars represent SD; P≥0.05; two tailed t test, n=8vs7). (B) qRT-PCR analysis of small intestine tissue from day-31 *Apc p53* and *Apc p53 Rac1b* mice for *Lyz1*, *Defa20* and *Muc2* (i), *Lgr5*, *Olfm4* and *Slc14a1* (ii) and *Axin2*, *Tcf7*, *Lef1* and *Ctnnb1* (iii) (error bars represent SD; P≥0.05; two tailed t test, n=3vs3). (C) Histological characterisation of *Apc p53* and *Apc* *p53 Rac1b* day-31 small intestine sections stained for H&E, Lysozyme, pH2AX and βcatenin. Scale bars are 100µm for H&E, pH2AX and β-Catenin IHC ,and 50µm for Lysozyme.

**
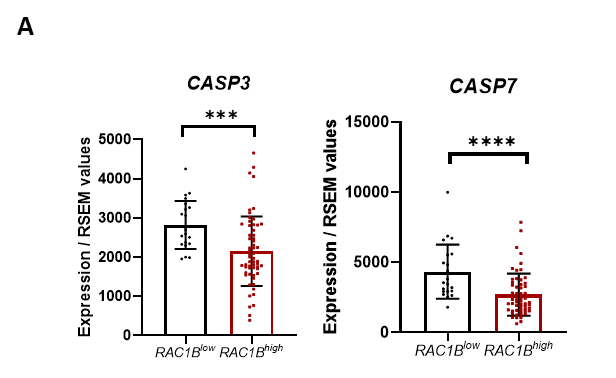
**

**Figure S3. Negative regulation of TGFβ signalling by *RAC1B* *in vivo*.** (A) Expression of CASPASE 3 (*CASP3*) and CASPASE 7 (*CASP7*) in *RAC1B^low^* and *RAC1B^high^* tumours in the COAD TCGA database (error bars represent SD; ***P<0.001, ****P<0.001; two tailed t test, n=22vs59).

| **Supplementary table 1: TGFβ pathway core genes (cbioportal.org, accessed 04/17/21)** |
| --- |
| TGFB1 |
| TGFB2 |
| TGFB3 |
| TGFBR1 |
| TGFBR2 |
| TGFBR3 |
| BMP2 |
| BMP3 |
| BMP4 |
| BMP5 |
| BMP6 |
| BMP7 |
| GDF2 |
| BMP10 |
| BMP15 |
| BMPR1A |
| BMPR1B |
| BMPR2 |
| ACVR1 |
| ACVR1B |
| ACVR1C |
| ACVR2A |
| ACVR2B |
| ACVRL1 |
| Nodal |
| GDF1 |
| GDF11 |
| INHA |
| INHBA |
| INHBB |
| INHBC |
| INHBE |
| SMAD2 |
| SMAD3 |
| SMAD1 |
| SMAD5 |
| SMAD4 |
| SMAD9 |
| SMAD6 |
| SMAD7 |
| SPTBN1 |
| TGFBRAP1 |
| ZFYVE9 |

**Supplementary Materials and Methods**

**Immunohistochemistry**

Mice intestines were harvested and flushed with cold PBS, opened longitudinally and fixed as “Swiss-roll” sections for 24 hours at 4ºC. PFA was used as a fixative for IHC procedures, while tissue was fixed on Methacarn (60% Methanol, 30% Chloroform and 10% Glacial acetic acid) for tumour and apoptotic bodies scoring. Tissue was automatedly embedded in paraffin using the Tissue-TeK VIP infiltration Processor (Sakura) and paraffin blocks were cut into sections of 5µm thickness with the microtome (Leica). Deparaffined sections were antigen retrieved (Citrate buffer, Thermo Fisher) and incubated with the following antibodies and concentrations: BrdU, 1:500 (Bioss, bs-0489H), cleaved Caspase 3, 1:800 (R&D, AF853), β-catenin, 1:50 (BD Biosciences, 610154), Lyz1, 1:1000 (DAKO, A009) and pH2AX, 1:100 (Cell Signalling, 4370S). Secondary HRP-tagged antibodies were used (anti-mouse or anti-rabbit Dako EnVision+ system). Slides were digitalised with the Nanozoomer Digital slide scanner (Hamamatsu) and analysed with the viewer software NDP.view2 (Hamamatsu). Tumour proliferation scoring and tumour area analysis was automatedly conducted with the QuPath software (qupath.github.io). However, normal crypt proliferation and tumour apoptosis was manually quantified. A minimum of five tumours were quantified per mouse and at least thirty full crypts per mouse were scored for normal tissue.

**Western blot**

Epithelial cell extractions and organoids were lysed with RIPA buffer (Sigma, R0278) supplemented with 1% of protease and phosphatase inhibitors (Sigma P8340 and P0044) and quantified with the BCA Protein Assay Kit (Pierce). 10µg of denaturated protein was run into a 4-12% Bris-Tris protein gel (NuPage, Thermo Fisher), transferred to a nitrocellulose membrane and incubated with the following antibodies and concentrations: RAC1B, 1:1000 (Millipore, 09-271), βACTIN, 1:5000 (BD, 610154), SMAD4, 1:1000 (Santa Cruz, sc-7966) and BIM, 1:1000 (Cell Signalling, 2933). Secondary HRP-tagged antibodies were used (anti-rabbit or anti-mouse IgG, Cell Signalling). Chemiluminescence signal was detected with the ECL Plus substrate (Thermo Scientific) and band densitometry was scored with ImageJ.

**RNA isolation**

Intestinal tissue and tumours were preserved on RNAlater (Sigma, R0901) at -80ºC until RNA isolation, and RNA from organoids and epithelial extractions was isolated on fresh. RNA extraction was performed with the Qiagen RNeasy Mini Kit (Qiagen) following manufacturer’s instructions. For tissue RNA extraction, tissue was firstly homogenised with stainless steel beads and the Qiagen Tissuelyser LT (Qiagen). DNA-free removal kit (Ambion/applied Biosystems, AM1906) was used for genomic DNA contamination removal and RNA concentration was quantified with the Nanodrop ND-100 spectrophotometer (Thermo Fisher).

**cDNA synthesis qRT-PCR**

RNA was reversed transcribed with the qScript cDNA SuperMix reagent (Quanta Bioscience, 95048-100). PCR protocol (incubation, DNA polymerization and enzyme deactivation) was as follows: 5min at 25ºC, 30min at 42ºC and 5min at 85ºC. For qRT-PCR, the PCR product was diluted 1:10. SYBR Master Mix (Life technologies, A25742) was used with 0.5µM of reverse and forward primer each. Samples were assessed in duplicate and β-actin was used as house-keeping gene for CT-value normalisation. The qRT-PCR protocol (pre-incubation, amplification and melting curve) was as follow: 10s at 95ºC, 44 cycles of 30s at 60ºC and 30s at 72ºC, and 0.5ºC intervals from 65ºC to 95ºC. The list of primers used is the following:

| **Gene** | **Forward primer (5’-3’)** | **Reverse primer (5’-3’)** |
| --- | --- | --- |
| Axin2 | TGACTCTCCTTCCAGATCCCA | TGCCCACACTAGGCTGACA |
| βActin | GTGACGTTGACATCCGTAAAGA | GCCGGACTCATCGTACTCC |
| Bim | GACAGAACCGCAAGGTAATCC | ACTTGTCACAACTCATGGGTG |
| Cdkn1a | CCTGGTGATGTCCGACCTG | CCATGAGCGCATCGCAATC |
| Cdkn2b | TCTGCAGCTGGATCTGGTCC | TCCTGAAAGGTAGAGGGCCC |
| Ctnnb1 | ATGGAGCCGGACAGAAAAGC | CTTGCCACTCAGGGAAGGA |
| Defa20 | TGTAGAAAAGGAGGCTGCAATAG | AGAACAAAAGTCGTCCTGAGC |
| hRAC1B | CAGTAAGGGAGCTGCAGTGG | GCGAAGAGTTTGTCCTCAACC |
| Lef1 | TGTTTATCCCATCACGGGTGG | CATGGAAGTGTCGCCTGACAG |
| Lgr5 | GAGTCAACCCAAGCCTTAGTATCC | CATGGGACAAATGCAACTGAAG |
| Lyz1 | GAGACCGAAGCACCGACTATG | CGGTTTTGACATTGTGTTCGC |
| mRac1b | TGTGGTAAAGATAGACCCTCC | CCCACGAGGATGATAGGAGT |
| Muc2 | AGGGCTCGGAACTCCAGAAA | CCAGGGAATCGGTAGACATCG |
| Olfm4 | GCCACTTTCCAATTTCAC | GAGCCTCTTCTCATACAC |
| Slc14a1 | TTAAAGTAGACCGGGGTGAAAAC | ACCCGTGACGTAGCCAAGTA |
| Smad4 | ACACCAACAAGTAACGATGCC | GCAAAGGTTTCACTTTCCCCA |
| Smad7 | GGCCGGATCTCAGGCATTC | TTGGGTATCTGGAGTAAGGAGG |
| Tcf7 | AGCTTTCTCCACTCTACGAACA | AATCCAGAGAGATCGGGGGTC |
| TGFβ1 | CTCCCGTGGCTTCTAGTGC | GCCTTAGTTTGGACAGGATCTG |

**TCGA analysis**

Classification of COAD TCGA tumours in RAC1B^low^ and RAC1B^high^ was done as previously described^17^. In brief, normal and tumour tissue data from the COAD dataset was downloaded from the TCGASpliceSeq web-based platform^45^ and tumours were divided based on their PSI value^46^. Expression of *TβRI, TβRII, SMAD4, CASP3* and *CASP7* was downloaded from a public study containing TCGA COAD RSEM-calculated reads of tumours^47^. Mutational status of SMAD4 and the mutational information of core TGFβ pathway genes was downloaded from CBioPortal (cbioportal.org). The list of the forty-three members of the TGFβ pathway analysis was generated by CBioPortal and it is included in the Supplementary Table 1. . Mutations in at least one of these genes were considered as “presence” of mutations in the TGFβ pathway.
